# Correlates of monoicy and dioicy in hornworts, the apparent sister group to vascular plants

**DOI:** 10.1186/1471-2148-13-239

**Published:** 2013-11-02

**Authors:** Juan Carlos Villarreal, Susanne S Renner

**Affiliations:** 1 Systematic Botany and Mycology, Department of Biology, University of Munich (LMU), Munich, Germany

**Keywords:** Chromosome counts, Sexual systems, Spore size, Trait correlation

## Abstract

**Background:**

Whether male and female gametes are produced by single or separate individuals shapes plant mating and hence patterns of genetic diversity among and within populations. Haploid-dominant plants (“bryophytes”: liverworts, mosses and hornworts) can have unisexual (dioicous) or bisexual (monoicous) gametophytes, and today, 68% of liverwort species, 57% of moss species, and 40% of hornwort species are dioicous. The transitions between the two sexual systems and possible correlations with other traits have been studied in liverworts and mosses, but not hornworts. Here we use a phylogeny for 98 of the 200 species of hornworts, the sister group to vascular plants, representing roughly equal proportions of all monoicous and all dioicous species, to test whether transitions in sexual systems are predominantly from monoicy to dioicy as might be expected based on studies of mosses. We further investigate possible correlations between sexual system and spore size, antheridium number, ploidy level, and diversification rate, with character selection partly based on findings in mosses and liverworts.

**Results:**

Hornworts underwent numerous transitions between monoicy and dioicy. The transition rate from dioicy to monoicy was 2× higher than in the opposite direction, but monoicous groups have higher extinction rates; diversification rates do not correlate with sexual system. A correlation important in mosses, that between monoicy and polyploidy, apparently plays a small role: of 20 species with chromosome counts, only one is polyploid, the monoicous *Anthoceros punctatus*. A contingency test revealed that transitions to dioicy were more likely in species with small spores, supporting the hypothesis that small but numerous spores may be advantageous for dioicous species that depend on dense carpets of gametophytes for reproductive assurance. However, we found no evidence for increased antheridium-per-chamber numbers in dioicous species.

**Conclusions:**

Sexual systems in hornworts are labile, and the higher number of extant monoicous species (60%) may be largely due to frequent transitions to monoicy.

## Background

**Figure 1:**
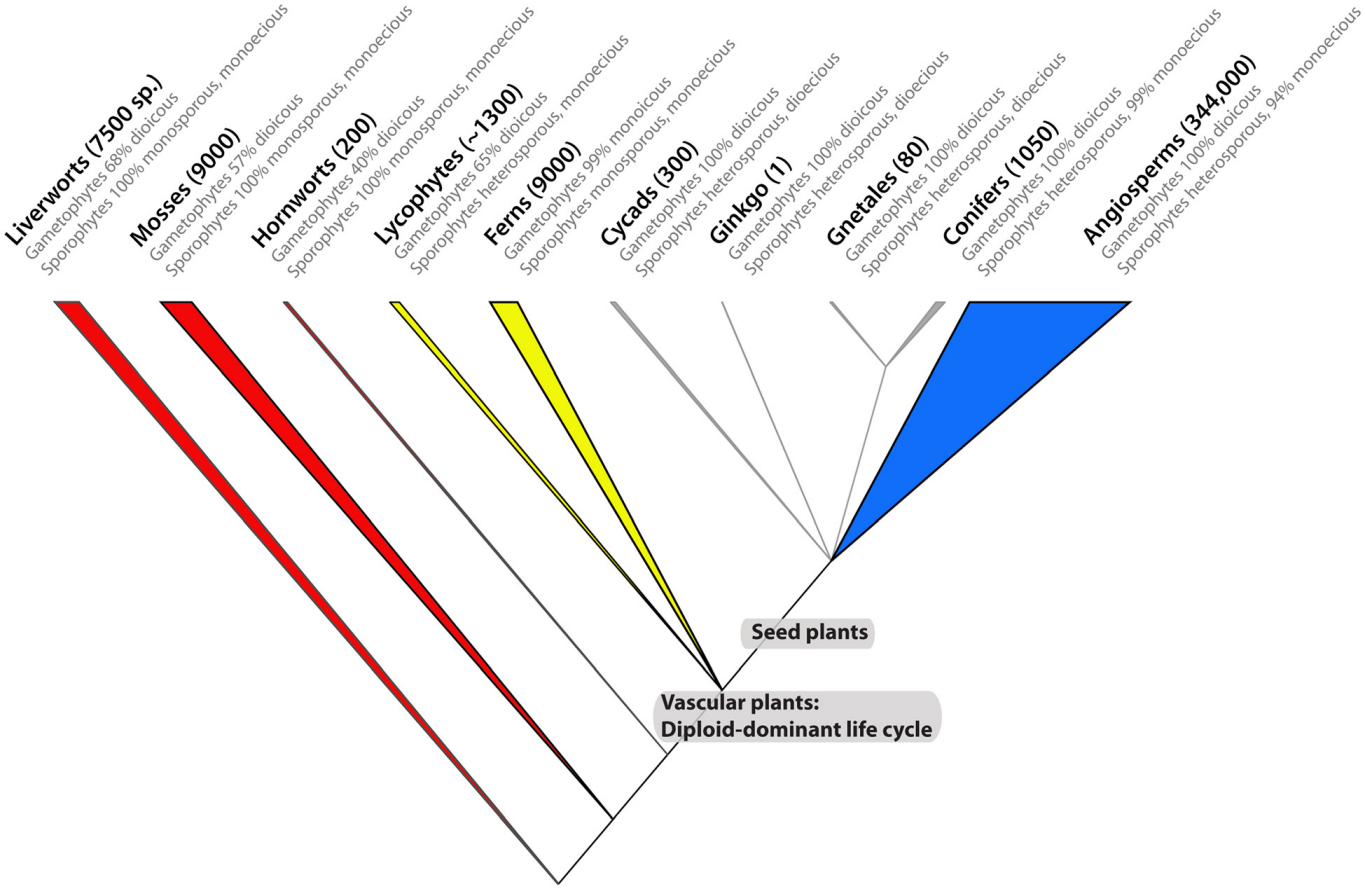
**Distribution of the four basic sexual systems of embryophytes.** The basic sexual system in embryophytes are illustrated namely, monoicy and dioicy in gametophytes (haploid organisms), and monoecy and dioecy in sporophytes (diploid organisms). Triangle width is proportional to species number and absolute species numbers are shown at the tips. The haploid-dominant lineages (liverworts, mosses, and hornworts) are shown in red triangles, the diploid-dominant lineages in yellow (lycophytes, ferns), gray (gymnosperms) and blue (angiosperms) triangles. Sources: Liverworts and mosses [2-5]; hornworts (this study); lycophytes and ferns [3,6]; http://www.rz.uni-karlsruhe.de/~db111/flora/ferns/index.php; seed plants ([7] and this study).

Understanding the evolution of plant sexual systems requires a phylogenetic background and a basic knowledge of plant life cycles. All embryophytes cycle between a haploid and a diploid life stage, and in the course of evolution, the diploid stage became the dominant phase in lycophytes, ferns and seed plants, while in liverworts, mosses, and hornworts the haploid stage is the dominant phase. This cycling has numerous implications, including for the evolution of plant sexual systems. There are four basic kinds of sexual systems, namely systems in which (i) the haploid stage produces archegonia and antheridia on each gametophyte (called monoicy); (ii) archegonia and antheridia are produced on separate gametophytes (dioicy); (iii) archegonia/embryo sacs and antheridia/microsporangia are produced on each sporophyte (monoecy); and (iv) archegonia/embryo sacs and antheridia/microsporangia are produced on separate sporophytes (dioecy; [1,2]; our Figure 1). Of these four systems, the two found in diploid-dominant plants, namely monoecy and dioecy, are not directly equivalent to the two found in haploid-dominant plants, namely monoicy and dioicy, because in haploid-dominants, the sporophyte is always monoicous, produces male and female spores (future gametophytes) in a 50:50 ratio, and is heterozygous at the sex locus, while in diploid-dominants, the sporophyte can become sexually specialized, then producing just one kind of spore (gametophyte), and be homozygous at the sex locus. This has evolved in cycads, *Ginkgo*, a few conifers (all Gnetales) and a few angiosperms (Figure 1). The frequencies of the four sexual systems in the major lineages of embryophytes vary dramatically.

Among the striking evolutionary changes along the phylogeny of embryophytes (Figure 1) is how the distribution of sexual systems changes between hornworts and vascular plants, their apparent sister group [8], partly because of the repeated evolution of heterospory, which entails sexually specialized gametophytes. Hornworts have 60% monoicous species and 40% dioicous ones, frequencies resembling those in liverworts and mosses, but the situation is completely different in ferns and fern-like lineages. In spite of their pivotal phylogenetic position, the evolution of monoicy and dioicy in hornworts has never been analyzed, probably because of the lack of a phylogenetic framework and outdated information on the presence or absence of monoicy and dioicy in the various genera. The directionality of change between monoicy and dioicy is therefore unknown. Here we use a modern hornwort phylogeny, based on plastid and mitochondrial loci and comprising 98 of the 200 species, and original data on the occurrence of monoicy and dioicy in the different species, to infer the minimal number of switches between monoicy and dioicy in this clade. We also test whether correlations between these two sexual systems and certain traits that have been detected in studies of liverworts and mosses, hold true in hornworts. We explain the background for these correlations in the next sections.

Sexual reproduction in dioicous hornworts, as in liverworts and mosses, depends on the availability of water and short distances between antheridia and archegonia, which is required for the motile sperm to fertilize the egg. Hornworts with separate-sexed gametophytes may therefore have a less assured reproduction than hornworts with cosexual gametophytes. However, intra-gametophyte mating is equivalent to the most extreme form of selfing, resulting in 100% homozygous progeny in a single generation. There is some evidence for selfing in monoicous mosses [9-11], but no data are available for hornworts. In spite of these expectations, monoicy and dioicy occur in roughly equal proportion in all three haploid-dominant embryophyte lineages (Figure 1). This raises the question of whether other traits precondition a lineage towards one of the two sexual systems. For example, in mosses, there appears to be a correlation between dioicy and small spores [12]. This became apparent in an analysis that used grafted phylogenies ([12]: Additional file 1: Table S1) to look at correlations between sexual system, spore size, seta length, and polyploidy. (Since the phylogeny was grafted, Crawford et al. [12] only analyzed correlations but did not infer the evolutionary direction of sexual system transitions). Dioicous mosses were more likely to have small spores if spore size was coded as a discrete character, but not if it was coded as a continuous character. The underlying cause of the possible correlation between spore size and monoicy was seen in the greater dispersal distance of small spores compared to large spores, which would compensate for higher local extinction rates of dioicous species (because of reproductive failure when sperm cannot reach egg cells).

In hornworts, spore sizes range from 18 μm diameter in *Leiosporoceros* up to >100 μm in the multicellular spores of *Dendroceros*[13]. If small spores are cheaper to produce than large ones they can be produced in larger numbers, which might be especially advantageous in dioicous species for which a dense spore shadow and dense population of gametophytes might provide reproductive assurance (because sperm would not have to swim far). The antheridia and archegonia of hornworts are embedded inside the thallus, and antheridium number varies from one up to 80 per chamber, with fairly narrow species-specific ranges [13,14]. Excluding possible animal vectors (such as mites), the flagellate sperm cells travel only a few centimeters [14-16]. Our initial expectation therefore was that dioicous hornworts would produce not only small spores, but also a higher number of antheridia per chamber than monoicous species to be able to produce more numerous sperm, again for reproductive assurance.

Besides testing for correlations between sexual system, spore diameter, and/or antheridium number, we were interested in whether there might be a correlation between polyploidy and sexual system. Hornworts usually have a low number of chromosomes, typically *n* = 5 (4 + U/V, the presumed sex chromosomes) in dioicous species and *n* = 5–6 in monoicous species, with few polyploid species [17]. Polyploidy is extremely rare in liverworts [18,19], but in mosses, polyploidy is common, and polyploid species tend to be monoicous ([2,12,20], and references therein). This is expected because following autopolyploidy, random pairing of the sex chromosomes will lead to both dioicous and monoicous offspring (depending on chromosome segregation), while following allopolyploidy, there will be only monoicous progeny, since after homeologous pairing each spore will contain a U chromosome and a V chromosome [2,20,21].

The species included in our phylum-wide phylogeny were selected to represent monoicous and dioicous species more or less in the proportion found across all hornworts, so as best to be able to answer the following questions: (i) Is dioicy or monoicy the more likely sexual system during the early phase of hornwort evolution? (ii) To what extent are shifts in sexual system correlated with shifts in spore size, antheridium number, and/or polyploidy? And (iii) is dioicy associated with lower diversification rates?

## Methods

### Taxon sampling, isolation of DNA, amplification, and sequencing

We sampled 98 of the 200 species of hornworts; Additional file 1: Table S1 provides a list of the sampled species with taxonomic author names, herbarium vouchers, and GenBank accession numbers for all sequences. Determination of plant material relied mostly on comparison with type material, but five of the sampled species are not yet formally described.

DNA isolation followed standard protocols. To deduce phylogenetic relationships we used the mitochondrial *nad*5-exon2, excluding an intron of ~950 nucleotides that is unique to *Leiosporoceros*, *Anthoceros*, *Folioceros* and *Sphaerosporoceros*[22] the plastid gene *rbc*L and portions of the trnK intron and the *mat*K gene contained within it (primers designed by Alan Forrest, Royal Botanical Garden Edinburgh). Total DNA from fresh, silica-dried or herbarium material was extracted with the Nucleo-Spin plant kit according to the manufacturer’s protocol (Macherey–Nagel, Düren, Germany). Primers and standard PCR protocols are listed in [23] except primers newly designed for *mat*K^a^. PCR products were cleaned using ExoSap-it (Affymetrix, Santa Clara, CA, USA), and sequencing reactions using Big Dye version 3.1 were run on an ABI 3130 capillary sequencer (Applied Biosystems, Perkin-Elmer, Wellesley, MA, USA), following manufacturers’ protocols. Sequence editing and alignment were carried out in Sequencher 4.7 (Gene Codes, Ann Arbor, MI, USA) and Geneious v. 5.6.6.

### Phylogenetic analysis

Combined phylogenetic analyses were performed under likelihood (ML) optimization and the GTR + G substitution model, using RAxML [24] with 500 bootstrap replicates. Bayesian analyses were conducted in MrBayes v. 3.2 [25], using the default two runs and four chains (one cold and three heated), with uniform priors on most parameters. Model parameters were unlinked, posterior probabilities of tree topologies were estimated from both partitions (the plastid and the mitochondrial data), and trees were sampled every 10000th generation. Burn-in and convergence were assessed using Tracer v. 1.5 [26]. Convergence was usually achieved after 4 × 10^6^ generations. We used 50% majority rule consensus trees to assess posterior probabilities for nodes of interest, and the 5000 trees with the highest likelihood were used for ancestral reconstructions. All analyses were run using the Cipres Science Gateway servers [27].

### Ancestral character reconstruction and correlated trait analyses

The sexual system (dioicous, state 0, or monoicous, state 1) of each species was scored from the literature or personal observations [28-33]. Sixty-five of the ~ 110 known monoicous species were sampled (~60%), and 33 of 54 known dioicous species (~61%). Trees were rooted on *Leiosporoceros*, the sole genus of the Leiosporocerotaceae, which based on outgroup-rooting is sister to all other hornworts [23]. This was preferable to outgroup comparison because two of the traits that we scored lack homologues in the vascular plants: Spore diameter (small, <35 μm, state 0, or large, >35 μm, 1) and antheridium number per chamber (low, <9, state 0, or high, >10, 1). Average mature spore diameters were taken from relevant literature [28-33]. The third trait, chromosome number, was taken from [34,35] and mapped onto the phylogeny.

Ancestral reconstruction relied on ML as implemented in Mesquite using the Markov 1- or 2- parameter models [36] and the highest likelihood tree from RAxML. To test the null hypothesis of equal transition frequencies between the two sexual systems, we performed a likelihood ratio test (LRT) that compared the likelihood of a 1-parameter model of equal transition rates (called q) with a 2-parameter, asymmetric model, which allows separate rates of transitions to monoicy and to dioicy [37]. Test significance was evaluated based on a *χ*2 distribution with 1 degree of freedom. We also reconstructed the ancestral conditions of spore size and antheridium number using a 1-parameter model or a 2-parameter model.

**Figure 2:**
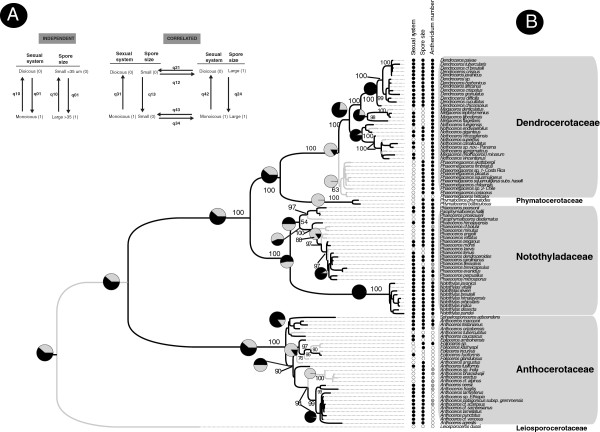
**Trait evolution and correlates of sexual systems in hornworts. 2A**. Models of trait evolution for sexual system and spore size. **(A)** Traits evolve independently from each other. The evolution of each trait is described by a forward rate q01 for shifts from state 0 to state 1, and a backward rate q10 for the reverse shift. **(B)** Traits can evolve in a correlated fashion, such that the rate of change in one trait depends on the background state of the other. A dual transition is not allowed. Joint evolution of two traits is thus described by an eight-rate model. **2B**. Maximum likelihood tree for 98 species of hornworts (from 3593 aligned nucleotides of plastid and mitochondrial DNA) with ancestral reconstruction of sexual systems (mapped as proportional likelihoods in pie diagrams above nodes). States for each terminal node are given for the following traits: sexual system (dioicous (0 ⁄ white) or monoicous (1 ⁄ black)). Large spore size (1 ⁄ black) or small spores (0 ⁄ white). Low antheridium number per chamber (1 ⁄ black) or large number (0 ⁄ white). Unknown character states are in dark grey. Numbers above branches represent bootstrap values.

To test for associations between sexual system and either spore diameter or antheridium number, we used a ML approach in the discrete module of BayesTraits [38]. The pair of traits was analyzed successively, using two models: a 4-rate model describing independent evolution of traits, and an 8-rate model describing correlated evolution (Figure 2A). Using the 5000 highest-likelihood trees from the Bayesian analyses, BayesTraits calculated likelihood scores for both of these models, showing how well the model fit the data. A LRT was then performed using the equation LR = -2 (L (dependent model) - L (independent model)) and a *χ*2 distribution, with four degrees of freedom (following [38]). If a trait association was barely significant we conducted 1000 simulations in Mesquite with 10 iterations for the likelihood search to obtain a *p* value; for trait associations that were significant, we tested hypotheses about conditional evolution and the temporal order of trait acquisition by comparing the likelihood estimates of two correlated-evolution models: one in which all transition rates were allowed to vary and one that constrained the two transition rates to be equal (Figure 2A). Contingency tests involved restricting one rate to be equal to the other, re-running the analysis, and performing a LRT of the two models, using a *χ*2 distribution with one degree of freedom [38].

### Character state-associated diversification

The diversification rate associated with each character state and the transition rates between character states have an impact on the distribution of binary traits [39,40]. We used the binary-state speciation and extinction (BiSSE) modelling approach to estimate transition rates among character states and character-associated diversification rates [39,40]. Our dataset includes 27 to 100% of the extant species in each of the hornwort genera (all of which have more than one species); sparse species sampling is a problem for all diversification modelling, and is also a problem in the present study. BiSSE model analyses were carried out in Mesquite using the diversitreeRpackage (http://www.zoology.ubc.ca/prog/diversitree/).

## Results and discussion

The combined plastid and mitochondrial alignment had 3593 nucleotide positions of which the *mat*K gene and portions of the trnK intron comprised 700–1000 positions; we excluded the intron in the *nad5* gene (Additional file 2: Matrix S1). Topologies from the individual partitions were congruent for most clades, although with less resolution in the tree obtained from the slow evolving *nad*5 alone. A ML tree from the combined data matrix has solid bootstrap support for most major hornwort clades except *Phaeomegaceros*, and its topology is consistent with previous phylogenetic reconstructions except for two novel findings: The nested position of *Folioceros* and *Sphaerosporoceros* within *Anthoceros* and the placement of two species of *Paraphymatoceros* (*P. hallii* and the type of the genus name, *P. diadematus*) as a clade nested within *Phaeoceros* along with the Californian *P. pearsonii* and *P. proskauerii* (Figure 2B*). Megaceros minarum* is embedded in *Nothoceros*, and the species will need to be transferred to that genus.

Figure 1 shows the distribution of the four basic sexual systems in embryophytes, namely dioicy/monoicy in haploid-dominants and dioecy/monoecy in diploid-dominant lineages (cf. *Introduction*). The evolution of heterospory in some ferns and the ancestor of all seed plants led to exclusively unisexual gametophytes, the ecological role of which is completely different from that of the free-living unisexual or bisexual gametophytes of the three haploid-dominant plant lineages. If early-diverging ferns and fern-like lineages had monoicous gametophytes, then the ancestral condition in hornwort gametophytes probably also was monoicous. The ancestral condition of the hornwort sporophyte is clear: All hornworts, ferns, and lycophytes have monoecious sporophytes.

Our ancestral state reconstruction of sexual systems under a 2-rate model suggests that monoicy may have predominated in the gametophytes of early hornworts (Figure 2B), and this sexual system is inferred as ancestral in *Dendroceros, Megaceros, Notothylas*, *Nothoceros* and Anthocerotaceae (Figure 2B). Dioicy is reconstructed as the ancestral condition for *Phymatoceros* and *Phaeomegaceros* (Figure 2B). Under ML optimization, the rate of transition from dioicy to monoicy was 98.38, and the backward rate was 56.25 (Table 1). Independent reversals from monoicy to dioicy were inferred for *Megaceros* (1 reversal)*, Nothoceros* (3) and *Phaeoceros* (1) (Figure 2B), with at least two transitions from dioicy to monoicy in *Folioceros* (Figure 2B).

**Table 1:** Reconstruction of trait evolution using a maximum likelihood approach

	**State**		**Models**			
**Trait**			**1-rate**	**2-rate**	**- log-likelihood- 1-rate**	**- log-likelihood- 2-rate**
Sexual system	Dioicous (0)	Monoicous (1)	62.08	98.38, 56.25	53.34	52.28
Spore size	Small (0)	Large (>35 μm) (1)	49.32	118.42, 53.92	50.84	49.30
Antheridium number	1-9 per chamber (0)	More than 10 (1)	7.66	5.83, 13.59	19.17	18.64

Log-likelihoods associated with the reconstruction of trait evolution for two competing explicit evolutionary models (see text for details) in hornworts. The two models (a single rate vs. a 2-rate model) were not significant different using a likelihood ratio test. The 2-rate values are given for a forward transition from state 0 to 1, followed by the backward rate (1 to 0).

Of the trait correlations tested, only spore size was correlated with transitions in sexual system: A contingency test revealed that transitions to dioicy were more likely in species with small spores as shown by the rate q31 of 199.72 compared with the rate q42 of 11.93 for transitions to dioicy in species with large spores (Table 2). An analysis using 5000 Bayesian trees indicated that the correlation between sexual system and spore diameter was barely significant (*χ*2 = 8.31, 4 df), while the 1000 simulations yielded a significant correlation (-log-likelihood -100.19 dependent model; -94.20 independent model, *p* = 0.01; Table 2). Evolutionary changes in antheridium number per chamber were unrelated to spore size. Large spores are the ancestral conditions in hornworts with at least 12 transitions to small spores and no reversals (Figure 2B). Many antheridia per chamber are found in the single species of *Leiosporoceros* and the ~80 species of *Anthoceros* (22 of them included here); few (1-8) antheridia per chamber are found in the Phymatocerotaceae/Dendrocerotaceae clade and some species of *Anthoceros*. For both traits, the unconstrained 2-rate model had a higher likelihood than the 1-rate model (Table 1), albeit not significantly so.

**Table 2:** Likelihood ratio values for analyses of trait correlation

**Trait**	**Change to large spores conditional upon sexual system state**	**Change to small spores conditional upon sexual system state**	**Change to monoicy conditional upon spore size state**	**Change to dioicy conditional upon spore size state**
Spore size	q12 = q34	q21 = q43	q13 = q24	q31 = q42
Rates	q12=70.44	q21 = 19.56	q13 = 277.5	q31 = 199.72
	q34=69.35	q43 = 45.59	q24 = 35.18	q42 = 11.93
L(U)	-92.92	-92.92	-92.92	-92.92
L(C)	-93.37	-93.58	-94.85	-95.81
×^2^	0.89	1.30	3.85*	5.77*

The meaning of the rate of transition between states (for example q12, q34) is shown in Figure 2A. Likelihood ratio values for analyses of correlations and tests of contingent evolution and temporal order between a life-history trait (spore size) and sexual system using *Discrete*. L (U) represents the likelihood of the unconstrained rate estimates, L (C) the likelihood when rates are set to be equal. Likelihood values were tested over a ×^2^ distribution with one degree of freedom. *P < 0.05. The only highly significant correlation was a gain of dioicy in lineages with small spores (×^2^= 5.77). A gain of monoicy in lineages with small spores was barely significant (×^2^= 3.85).

Bias in the estimation of transition rates in sexual systems is introduced when the sexual systems experience different rates of diversification [39,40]. Our BiSSE analyses, however, showed that while the transition rate from dioicy to monoicy was 4.2 higher than *vice versa* (q01 = 120.00; q10 = 28.17), the speciation rates in monoicous and dioicous clades were similar (λ_0_ = 265.34; λ_1 =_ 275.93). Monoicous groups accordingly had a higher extinction rate (μ_0_ = 182.49; μ_1_ = 294.11). The slightly higher number of extant monoicous species (60% vs. 40% dioicous species) may thus be entirely due to frequent transitions to monoicy.

In species with genetic sex determination, transition to monoicy may follow genome duplication, because genome doubling may disrupt the strict segregation of the male and female sex chromosomes (*Introduction)*. Only twenty species of hornworts have had their chromosomes counted, some repeatedly, resulting in 65 counts [34,35]. Sixteen of the twenty species are represented in our phylogeny (Additional file 3: Figure S1). So far, a single natural polyploid has been reported, the monoicous *Anthoceros punctatus*[17]. This fits the expected association of monoicy and polyploidy, but is insufficient to decide whether such an association holds across hornworts.

Our finding that lineages with small spores transition to dioicy more readily than those with large spores matches the situation in mosses, although in that clade this was only true if spore diameter was coded as a discrete character, but not when it was coded as a continuous character [12]. Crawford et al. [12] argued that evolutionary transitions to separate sexes might be easier if sporophytes produce small spores that might travel further. However, release height and wind speed probably override the importance of spore diameter in determining dispersal range [41], and it is also unclear why further dispersal might be more beneficial for dioicous species than monoicous ones. In hornworts, the sporophytes elongate by means of a basal meristem, with mature sporangium length varying from 2–3 mm in some species of *Notothylas* to >10 centimeters in species of *Anthoceros*, *Megaceros*, and *Nothoceros*. A more plausible explanation for the correlation between having small spores and transition to dioicy therefore is a resource allocation trade-off, such that larger numbers of spores can only be produced when each spore has a small diameter. Once a lineage has small spores, this may facilitate its transition to separate sexes because female thalli producing more numerous spores create a denser spore shadow, permitting sperm to more easily reach thalli of the opposite sex. Data on spore number per capsule so far are only available for *Anthoceros agrestis* (with large spores) [42], and experiments are required to test the above-proposed hypothesis.

In monoicous species of hornworts, the antheridia tend to develop earlier than the archegonia [14], minimizing intra-gametophyte fertilization and perhaps reducing inbreeding and the accompanying selection for dioicy. Lastly and importantly, our study of sexual system switches in hornworts, together with the data on such switches available for liverworts [18,19] and mosses [43,44], makes clear that suggestions of dioicy being ancestral in “bryophytes” (at the time assumed to be a monophylum) and of monoicy being the derived sexual system [2] need to be put to rest. Instead, sexual systems in the three lineages of haploid-dominant land plants are highly labile, and there is no single preferential direction. Given the under-sampling in all phylogenies used in studies of sexual system evolution to date, back and forth transitions between dioicy and monoicy probably are still underestimated. Quantitative and experimental studies of the apparent trade-off between spore diameter, spore number, and spore shadows are much needed.

## Conclusions

A phylogeny for 98 of the 200 species of hornworts, representing roughly equal proportions of their monoicous and dioicous species, implies many shifts between monoicy and dioicy, terms that refer to the sexual systems of haploid tissues (compare Figure 1 for the distribution of the four main sexual systems in land plants). Different from mosses, the transition rate from dioicy to monoicy in hornworts exceeds that in the opposite direction, while diversification rates do not differ with sexual system. Trait correlation analyses revealed that transitions to dioicy are more likely in species with small spores, while the opposite is not the case. If smaller spores can be produced in larger numbers, smaller-spored species may enjoy denser spore shadows, ensuring successful fertilization because of shorter distances between male and female gametophytes. However, we found no evidence of increased antheridium-per-chamber numbers in dioicous species. A correlation between monoicy and polyploidy apparently plays a small role in hornworts.

## Endnote

^a^(http://api.ning.com/files/MUfcgT2TON39NNX3xqXMe1AuDyInYb-Bucg5pdmTT9nuFWp27BtbiJKU1bsG9aU8t5OEjIIw8QJqaMs4qIlnbQ*xZ1mKSP1x/Hornwort_matK_RBGE_PROTOCOLv1.0.pdf).

## Competing interests

The authors declare that they have no competing interests.

## Authors’ contributions

JCV performed DNA sequencing and alignment; JCV analyzed data; JCV and SSR wrote the paper; JCV and SSR designed research; both authors read and approved the final manuscript.

## Supplementary Material

Additional file 1: Table S1 Voucher and GenBank information. List of species used in this study including their author names, herbarium vouchers, and GenBank accession numbers for all sequences. Click here for file

Additional file 2 **Matrix S1 DNA alignment, morphological matrix and tree used in the study.** NEXUS file of the alignment for 98 species of hornworts (from 3593 aligned nucleotides of plastid and mitochondrial DNA), a morphological matrix and maximum likelihood tree. Click here for file

Additional file 3: Figure S1 Chromosome number and evolution of sexual systems in hornworts. Maximum likelihood tree for 98 species of hornworts (from 3593 aligned nucleotides of plastid and mitochondrial DNA) with ancestral reconstruction of sexual systems (mapped as proportional likelihoods in pie diagrams above nodes). States for each terminal node are given for the following traits: sexual system (dioicous (0 ⁄ white) or monoicous (1 ⁄ black)). Chromosome counts are mapped onto the tree for monoicous species (black stars) and dioicous species (grey stars). *Anthoceros punctatus* has the karyotype of a monoicous species and is a natural polyploid. Inset: The karyotypes of the dioicous *Phymatoceros bulbiculosus* from Portugal and of the monoicous *Nothoceros vincentianus* from Peru (listed as *Megaceros sp*. in the original paper, later identified by Proskauer as *Megaceros vincentianus*). Dioicous species typically have four chromosomes and one U/V sex chromosomes (the U chromosome slightly larger). Monoicous species typically have 5 large chromosomes with numerous (1–5) “m” or accessory chromosomes that can vary within a single gametophyte (modified from [17], all chromosomes drawn at the same scale). Click here for file
