# Supplementary material for: Correlates of monoicy and dioicy in hornworts, the apparent sister group to vascular plants
Source: BMC Evol Biol. 2013 Nov 2;13:239. doi: 10.1186/1471-2148-13-239 (PMC4228369; doi:10.1186/1471-2148-13-239)
Supplement: Additional file 1: Table S1 — Voucher and GenBank information. List of species used in this study including their author names, herbarium vouchers, and GenBank accession numbers for all sequences. [file 1471-2148-13-239-S1.doc]

Table S1. List of species used in this study including their author names, herbarium vouchers, and GenBank accession numbers for all sequences, new sequences are in bold.

| **Species name** | **Voucher (Herbarium)** | **Source** | ***rbc*L** | ***nad*5** | ***trnK-matk*** |
| --- | --- | --- | --- | --- | --- |
| *Anthoceros agrestis* Paton | Doyle 11342 (M) | Duff et al. (2007), this study | DQ845682.1 | DQ845731.1 | --------------- |
| *Anthoceros alpinus* Steph. | Duckett IW110 (M) | This study | **KF482268** | **KF482243** | **KF482215** |
| *Anthoceros angustus* Steph. | Zhang 7704 (SZG), no voucher for the genome, probably Yoshinaga s.n. | Li et al. (2012) | AB086179.1 | JF815561.1 | AB086179.1 |
| *Anthoceros bharadwajii* Udar & Asthana | Chantanaorrapint 229 (PSU) | This study | **KF482267** | **KF482242** | **KF482214** |
| *Anthoceros caucasicus* Steph. | Garcia s.n. (M) | Villarreal & Renner (2012), this study | JX872419 | JX872453 | **KF482294** |
| *Anthoceros* cf. *sambesianus* Steph. | Serousiaux s.n. (M) | Villarreal & Renner (2012), this study | JX872425 | JX872460 | **KF482295** |
| *Anthoceros* cf. *scariosus* Aust. | Villarreal 1217 (M) | Villarreal & Renner (2012), this study | JX872420 | JX872454 | **KF482296** |
| *Anthoceros cf. venosus* Lindenb et Gottsche | Salazar et al. 20654 (PMA) | Villarreal & Renner (2012), this study | JX872428 | JX872463 | **KF482297** |
| *Anthoceros erectus* Kash. | Chantanaorrapint 212, 550 (PSU) | This study | **KF482269** | -------------- | **KF482216** |
| *Anthoceros fragilis* Steph. | R. Lovatt & A. Holland TH 9821 (CANB) | This study | **KF482270** | **KF482244** | **KF482298** |
| *Anthoceros fusiformis* Aust. | Doyle 11347 (ABSH, M) | Duff et al. (2007), this study | DQ845677 | DQ845727.1 | **KF482217** |
| *Anthoceros lamellatus* Steph. | Duckett s.n. (ABSH); Rincon s.n. (M) | Duff et al. (2007); Villarreal & Renner (2012); this study | DQ845679 | JX872455 | **KF482299** |
| *Anthoceros laminiferus* Steph. | Duckett, s.n. (ABSH); Slack 211056 (M) | Duff et al. (2007), this study | **KF482271** | DQ845728.1 | **KF482300** |
| *Anthoceros macounii* M.A. Howe | Faubert s.n. (QFA, M) | Villarreal & Renner (2012), this study | JX872421 | JX872456 | **KF482301** |
| *Anthoceros neesii* Prosk. | Manzke s.n. (M) | Villarreal & Renner (2012), this study | JX872422 | JX872457 | **KF482302** |
| *Anthoceros orizabensis* (Steph.) Hässel | Villarreal 770 (M) | Villarreal & Renner (2012), this study | JX872423 | JX872458 | **KF482303** |
| *Anthoceros patagonicus* Hässel subsp. *gremmenii* J.C. Villarreal et al. | Gremmen 2005-T028 (F) | Villarreal & Renner (2012), this study | JX872424 | JX872459 | **KF482304** |
| *Anthoceros punctatus* L. | Sergio s.n. (LISU, M), Chamberlain & Kungu s.n (E) | Duff et al. (2007), this study | **KF482272** | DQ845730 | **KF482305** |
| *Anthoceros sp.1. Ethiopia* | Hylander 4504 (M) | This study | **KF482273** | **KF482245** | **KF482306** |
| *Anthoceros sp.2- India* | Duckett IW57 (BM) | This study | **KF482274** | ---------- | **KF482218** |
| *Anthoceros tristanianus* J.C. Villarreal et al. | Villarreal 1032 (M) | Villarreal & Renner (2012), this study | JX872426 | JX872461 | **KF482219** |
| *Anthoceros tuberculatus* Lehm. & Lindenb. | Villarreal & Rodríguez 857 (CONN) | Villarreal & Renner (2012), this study | JX872427 | JX872462 | **KF482307** |
| *Dendroceros aff. cucullatus* Steph. | Chantanaorrapint 1623 (PSU) | This study | **KF482275** | **KF482251** | **KF482308** |
| *Dendroceros africanus* Steph. | Shevock 39829 (M) | This study | **KF482276** | **KF482252** | **KF482220** |
| *Dendroceros borbonicus* Steph. | Theo Aarts 153-51 (BR) | This study | **KF482277** | **KF482253** | **KF482221** |
| *Dendroceros cf. breutelii* Nees | Buck 51344 (NY) | Villarreal & Renner (2012), this study | JX872429 | JX872464 | **KF482309** |
| *Dendroceros* *chicoraceus* (Mont.) Gottsche | Larraín 31162 (CONC) | Villarreal & Renner (2012), this study | JX872430 | JX872465 | **KF482310** |
| *Dendroceros crispatus* (Hook.) Nees | Cargill 28 (CANB), Paterson s.n. B570 (CANB) | Duff et al. (2007), this study | AY463048 | DQ845708.1 | **KF482311** |
| *Dendroceros crispus*  (Sw.) Nees | Villarreal 1296 (M) | This study | JX885633 | **KF482254** | **KF482312** |
| *Dendroceros difficilis* Steph. | Von Konrat s.n. (FM) | Villarreal et al. (2012), this study | HM056148.1 | JX872466 | JN559927.1 |
| *Dendroceros granulatus* (Mitt.) Steph. | Duckett sn. (ABSH), Cargill DAM-KI 130 (CANB) | Duff et al. (2007); this study | AY463049 | ---------- | **KF482222** |
| *Dendroceros javanicus* (Nees) Nees | Buchbender, s.n. (CONN) | Villarreal & Renner (2012), this study | JX872431 | JX872467 | **KF482313** |
| *Dendroceros paivae* Garcia et al. | Garcia, C., ST 125 (LISU 237201) | Villarreal & Renner (2012), this study | JX872432 | JX872468 | **KF482314** |
| *Dendroceros* sp. | Gradstein 12106 (DUKE) | This study | **KF482278** | KF482255 | **KF482223** |
| *Dendroceros tubercularis* Hatt. | Shevock 39039 (M) | This study | **KF482279** | KF482256 | **KF482224** |
| *Folioceros amboinensis* (Schiffn.) Steph. | Chantanaorrapint 2493 (PSU) | This study | **KF482280** | KF482246 | **KF482315** |
| *Folioceros sp.* | Peng_20111016-3 (HSNU) | This study | **KF482284** | KF482249 | **KF482227** |
| *Folioceros fuciformis* (Mont) D.C. Bharadwaj. | Cargill, D.C. s.n. (CANB); Gradstein 12350 (M) | Duff et al. (2007), this study | **KF482281** | [DQ845726.1](http://www.ncbi.nlm.nih.gov/nucleotide/139525600?report=genbank&log$=nucltop&blast_rank=58&RID=WCEPHDCN012) | **KF482316** |
| *Folioceros glandulosus* (Lehm. & Lindenb.) D.C. Bharadwaj | Zhang 7704  (SZG) | Li et al. (2012), this study | JF815573.1 | JF815563.1 | ------------- |
| *Folioceros kashyapii* Udar & Srivastava | Peng_20111015-85 (HNSU) | This study | **KF482283** | **KF482248** | **KF482226** |
| *Folioceros incurvus* (Steph.) D.C. Bharadwaj | Shevock_39742 (M) | This study | **KF482282** | **KF482247** | **KF482225** |
| *Leiosporoceros dussii* (Steph.) Hässel | Villarreal & Araúz 851, 1285 (PMA) | Duff et al. (2007), this study | AY463052.1 | AY894803.1 | **KF482228** |
| *Megaceros sp.* | Lewington 1121 (CANB) | This study | **KF482285** | **KF482257** | **KF482229** |
| *Megaceros flagellaris* (Mitt.) Steph. | D.C. Cargill 885 (CANB) | Villarreal et al. (2010), this study | GQ845371 | GQ845372.1 | JN559929 |
| *Megaceros leptohymenius* (Hook. f. & Tayl.) Steph. | Duckett 3N-117; 3N36 (M) | Villarreal et al. 2010, this study | JX885636 | **KF482258** | **KF482230** |
| *Megaceros tjibodensis* Campb. | Ducket IE52 (M) | This study | **KF482286** | **KF482259** | **KF482231** |
| *Megaceros minarum* (Nees) Steph. | Cargill & Prieto 2625 (CANB) | (Villarreal & Renner 2012), this study | JX872433 | JX872471 | **KF612916** |
| *Nothoceros aenigmaticus* (R.M. Schust.) J.C.Villarreal & McFarland | Villarreal & McFarland 935 (CONN) | Villarreal et al. 2012; this study | GQ504731 | GQ504735 | **KC285889** |
| *Nothoceros canaliculatus* (Pagan) J.C. Villarreal et al. | Lepiz, s.n. (M) | Villarreal et al. 2012; this study | HM056176.1 | JX872470 | JN559952.1 |
| *Nothoceros sp. nov.* | Villarreal & Varela 584 (PMA) | Villarreal et al. 2010; Villarreal et al. 2012; this study | GQ504732 | GQ504736 | JN559949.1 |
| *Nothoceros endiviifolius* (Mont). J. Haseg. | Duckett, s.n. (CONN) | Duff et al. (2007); this study | DQ845645 | GQ504737 | JN559930.1 |
| *Nothoceros fuegiensis* (Steph.) J.C.Villarreal | Goffinet 9527 (CONN) | Villarreal et al. (2010) | HM056156 | DQ097162 | JN559934 |
| *Nothoceros giganteus* (Lehm. & Lindenb.) J. Haseg. | Engel & Von Konrat 27407 (M) | Villarreal et al. (2010) | HM056154 | DQ845709 | JN559932 |
| *Nothoceros renzagliensis* J.C. Villarreal et al. | Villarreal et al. 1080 (COL) | Villarreal et al. (2012); this study | HM056162.1 | JX872472 | JN559940 |
| *Nothoceros superbus* J.C.Villarreal et al. | Salazar et al. 20676 (PMA) | Villarreal et al. (2012); this study | HM056172.1 | JX872473 | **KF482317** |
| *Nothoceros vincentianus* (Lehm. & Lindenb.) J.C.Villarreal | Villarreal & Rodrıíguez 840 (CONN); Villarreal et al. 641 (ABSH) | Villarreal et al. (2012); this study | HM056171 | DQ845711 | JN559943 |
| *Notothylas breutelii* (Gottsche) Gottsche | Krayeski, s.n. cultured (ABSH) | Duff et al. (2007), this study | AY463054 | DQ845719 | **KF482318** |
| *Notothylas dissecta* Steph | Araúz et al. 798 (M, PMA) | (Villarreal & Renner 2012), this study | JX872434 | JX872474 | **KF482319** |
| *Notothylas himalayensis* Udar & Singh | Ducket IW56 (M) | This study | **KF482287** | **KF482260** | **KF482232** |
| *Notothylas indica* Kashyap | Ducket IE38 (M) | This study | **KF482288** | **KF482261** | **KF482233** |
| *Notothylas javanica* (Sande Lac.) Gottsche | Villarreal 806 (PMA), Villarreal 1311 (M) | Duff et al. (2007), this study | JX885638 | DQ845720 | **KF482320** |
| *Notothylas levieri* Steph. ex Schiffn. | Long 30668 (E) | (Villarreal & Renner 2012), this study | JX872436 | JX872475 | **KF482234** |
| *Notothylas orbicularis* (Schwein.) Sull ex A. Gray | Villarreal et al. 1302 (M) | Duff et al. (2007), this study | JX885639 | **KF482262** | ---------- |
| *Notothylas pandei* Udar & Chandra | Chantanaorrapint 1666 (PSU) | This study | **KF482289** | **KF482263** | **KF482235** |
| *Notothylas vitalii* Udar & Singh | Gradstein s.n. (PER) | (Villarreal & Renner 2012), this study | JX872437 | JX872476 | -------- |
| *Paraphymatoceros diadematus* Hässel | Larraín 34069 (CONC) | (Villarreal & Renner 2012), this study | JX872438 | JX872477 | **KF482321** |
| *Paraphymatoceros hallii* (Aust.) Hässel | Doyle 11363 (ABSH) | Duff et al. (2007); this study | DQ845670 | JX872478 | **KF482236** |
| *Phaeoceros* *pearsonii* (M.A. Howe) Prosk. | Doyle s.n (M) | Duff et al. (2007), this study | DQ845668.1 | AY894802.1 | **KF482322** |
| *Phaeoceros* *proskauerii* Stotler et al. | Doyle 11339 (ABSH) | This study | EU283415.1 | JX872479 | **KF482323** |
| *Phaeoceros brevicapsulus* (Steph.) Hässel | Queralta, s.n. (M) | Villarreal & Renner (2012), this study | JX872439 | JX872480 | --------------- |
| *Phaeoceros carolinianus* (Michx.) Prosk. | Shevock 39775 (M) | This study | **KF482290** | **KF482264** | **KF482237** |
| *Phaeoceros cf. bolussi* (Sim) S. Arnell | Hedderson 16894 (BOL) | Villarreal & Renner (2012), this study | JX872440 | JX872481 | **KF482324** |
| *Phaeoceros dendroceroides* (Steph.) Hässel | Villlarreal 1305 (M) | this study | **KF482291** | **KF482265** | **KF482325** |
| *Phaeoceros engellii* Cargill & Fuhrer | Cargill & Fuhrer 1015 (CANB) | (Villarreal & Renner 2012), this study | JX872441 | JX872482 | **KF482326** |
| *Phaeoceros evanidus* (Steph.) Cargill & Fuhrer | Cargill 875 (CANB) | (Villarreal & Renner 2012), this study | JX872442 | JX872483 | **KF482327** |
| *Phaeoceros flexivalvis* (Nees & Gottsche) Hässel | Villarreal 863 (M) | (Villarreal & Renner 2012), this study | JX872443 | JX872484 | **KF482238** |
| *Phaeoceros himalayensis* (Kash.) Prosk. | Long 30423 (E) | (Villarreal & Renner 2012), this study | JX872444 | JX872485 | **KF482239** |
| *Phaeoceros inflatus* (Steph.) Hässel | Cargill & Fuhrer 474 (CANB) | (Villarreal & Renner 2012), this study | JX872445 | JX872486 | **KF482328** |
| *Phaeoceros laevis* (L). Prosk. | Sergio, s.n. (LISU) | Duff et al. (2007), this study | DQ845673 | DQ845721 | **KF482240** |
| *Phaeoceros microsporus* (Steph.) Hässel | Villarreal 725 (M) | (Villarreal & Renner 2012), this study | JX872446 | JX872487 | **KF482329** |
| *Phaeoceros minutus* (Steph.) S. Arnell | Hedderson 16879 (BOL) | (Villarreal & Renner 2012), this study | JX872447 | JX872488 | **KF482330** |
| *Phaeoceros mohrii* (Aust.) Hässel | Doyle 11341 (M) | Duff et al. (2007), this study | DQ845672 | DQ845724 | **KF482331** |
| *Phaeoceros oreganus* (Aust.) Hässel | Doyle 11382 (M) | (Villarreal & Renner 2012), this study | DQ845661 | JX885644 | **KF482332** |
| *Phaeoceros perpusillus* S. Chantanaorrapint | Chantanaorrapint 1551 (PSU) | This study | **KF482292** | **KF482266** | **KF482333** |
| *Phaeoceros tenuis* (Spruce) Hässel | Ibarra Morales #17 ( FCME) | (Villarreal & Renner 2012), this study | JX872448 | ------------- | **KF482334** |
| *Phaeomegaceros chiloensis* (Steph.) J.C.Villarreal | Larraín 34061 (CONC) | (Villarreal & Renner 2012), this study | JX872449 | JX872489 | --------------- |
| *Phaeomegaceros coriaceus* (Steph.) Duff et al. | Glenny 9757 (CONN) | (Villarreal & Renner 2012), this study | JX872450 | JX872490 | **KF482335** |
| *Phaeomegaceros fimbriatus* (Gottsche) Duff et al. | Villarreal 779 (ABSH); Villarreal et al. 881 (CONN) | Duff et al. (2007), this study  Villarreal et al. (2012), this study | HM056149 | DQ845716 | **JN559928** |
| *Phaeomegaceros hirticalyx* (Steph.) Duff et al. | Duckett s.n. (ABSH, M) | Duff et al. (2007), this study | AY463043 | DQ845713 | **KF482336** |
| *Phaeomegaceros plicatus* (Mitt) J.C. Villarreal, Engel & Vana | Gremmen T07-1097 (F) | (Villarreal & Renner 2012), this study | JX872451 | JX872491 | **KF482337** |
| *Phaeomegaceros skottsbergii* (Steph.) Duff et al. | Cuvertino s.n. (SGO) | Duff et al. (2007), this study | DQ845659 | DQ845715 | --------------- |
| *Phaeomegaceros sp. nov. 1* | Villarreal et al. 871 (M) | (Villarreal & Renner 2012), this study | JX872452 | JX872492 | **KF482338** |
| *Phaeomegaceros sp. nov. 2* | Duckett s.n. (ABSH) | Duff et al.(2007), this study | DQ 845651 | DQ845714 | **KF482339** |
| *Phaeomegaceros squamuligerus* (Spruce) J.C. Villarreal | Jofre s.n (CONN) | Villarreal et al. (2010), this study | HM038430 | HM038432 | **KF482340** |
| *Phaeomegaceros squamuligerus subsp. hasselli* J.C. Villarreal | Goffinet 7106 (CONN) | Villarreal et al. (2010), this study | HM038429 | HM038431 | **KF482341** |
| *Phymatoceros phymatodes* (M.A. Howe) Duff et al. | Doyle s.n, Doyle11480 (ABSH, M) | Duff et al. (2007), this study | DQ845660.1 | DQ845717.1 | **KF482342** |
| *Phymatoceros bulbiculosus* (Brot.) Stotler et al. | Sergio s.n. (LISU), | Duff et al. (2007), this study | DQ268978.1 | DQ097163.1 | **KF482241** |
| *Sphaerosporoceros adscendens* (Lehm. & Lindenb.) Hässel | Hays 4201-3 (M) | This study | **KF482293** | **KF482250** | --------------- |

References

1. Duff RJ, Villarreal JC, Cargill DC, Renzaglia KS: **Progress and challenges toward developing a phylogeny and classification of the hornworts**. *Bryologist* 2007, 110: 214-243.
2. Kugita M, Kaneko A, Yamamoto Y, Takeya Y, Matsumoto T, Yochinaga K: **The complete nucleotide sequence of the hornwort (*Anthoceros formosae*) chloroplast genome: Insight into the earliest land plants**. *Nucleic Acids Research* 2003, *31*: 716-721.
3. Li J, Zhang L, Zhou L: **Phylogenetic position of the genus *Hattorioceros* (Anthocerotophyta)**. *Taxon* 2012, 60: 1633-163.
4. Villarreal JC, Goffinet B, Duff RJ, Cargill DC: **Phylogenetic delineation of *Nothoceros* and *Megaceros* (Dendrocerotaceae)**. *Bryologist* 2010, 113: 106-113.
5. Villarreal JC, Renner SS: **Hornwort pyrenoids, a carbon-concentrating mechanism, evolved and were lost at least five times during the last 100 million years**. *Proc Natl Acad Sci, USA* 2012, 109: 18873-18878.
