# Supplementary figures and images for: Correlates of monoicy and dioicy in hornworts, the apparent sister group to vascular plants
Source: BMC Evol Biol. 2013 Nov 2;13:239. doi: 10.1186/1471-2148-13-239 (PMC4228369; doi:10.1186/1471-2148-13-239)

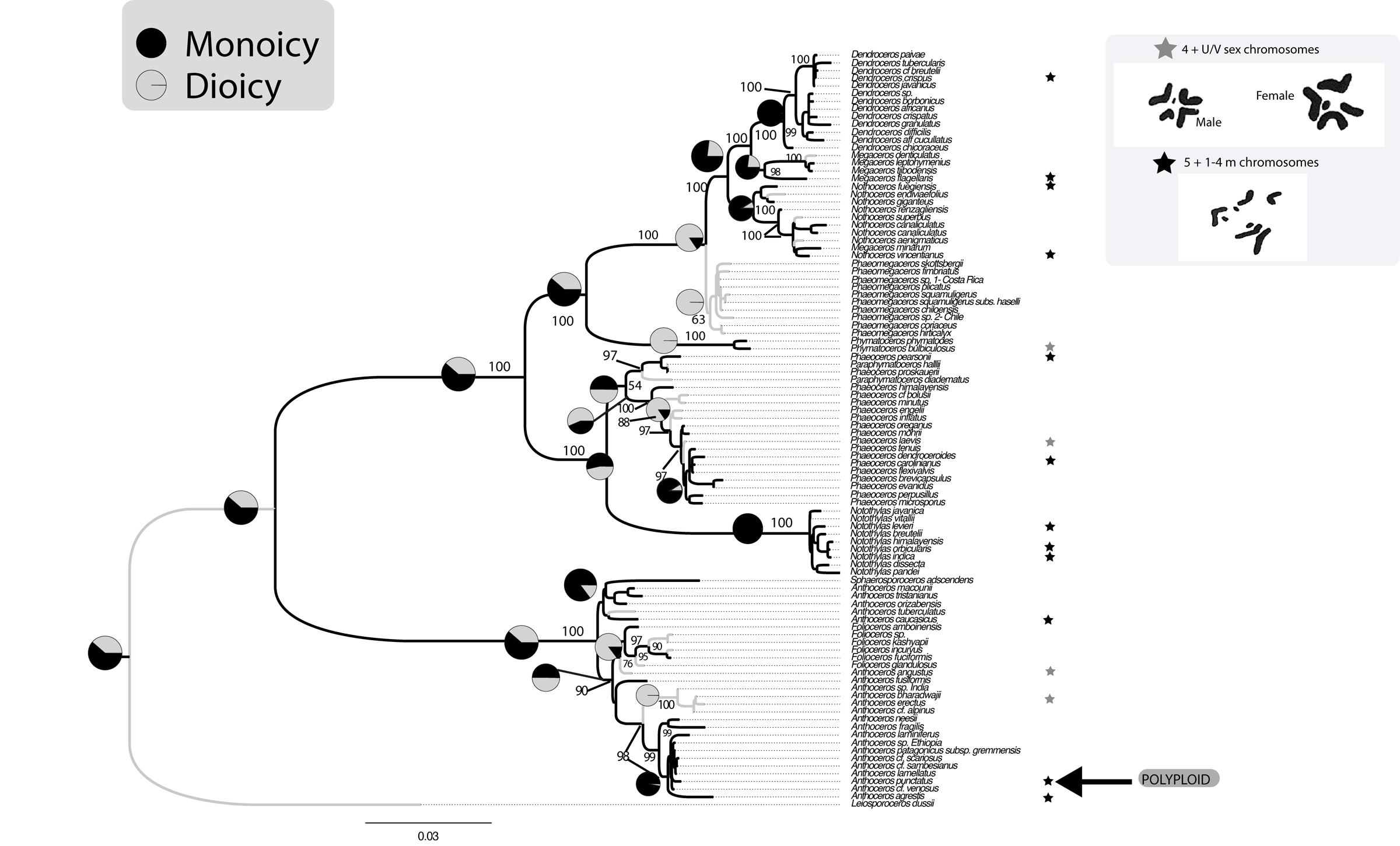

Supplement: Additional file 3: Figure S1 — Chromosome number and evolution of sexual systems in hornworts. Maximum likelihood tree for 98 species of hornworts (from 3593 aligned nucleotides of plastid and mitochondrial DNA) with ancestral reconstruction of sexual systems (mapped as proportional likelihoods in pie diagrams above nodes). States for each terminal node are given for the following traits: sexual system (dioicous (0 ⁄ white) or monoicous (1 ⁄ black)). Chromosome counts are mapped onto the tree for monoicous species (black stars) and dioicous species (grey stars). Anthoceros punctatus has the karyotype of a monoicous species and is a natural polyploid. Inset: The karyotypes of the dioicous Phymatoceros bulbiculosus from Portugal and of the monoicous Nothoceros vincentianus from Peru (listed as Megaceros sp. in the original paper, later identified by Proskauer as Megaceros vincentianus). Dioicous species typically have four chromosomes and one U/V sex chromosomes (the U chromosome slightly larger). Monoicous species typically have 5 large chromosomes with numerous (1–5) “m” or accessory chromosomes that can vary within a single gametophyte (modified from [17], all chromosomes drawn at the same scale). [file 1471-2148-13-239-S3.tiff]
